# Deciphering Complexity: Atypical Hemolytic Uremic Syndrome Unraveled in the Wake of Elective Hip Arthroplasty

**DOI:** 10.7759/cureus.68690

**Published:** 2024-09-05

**Authors:** Gautam N Bedi, Tushar Sontakke, Smruti A Mapari, Rucha Sawant, Nikhil Reddy

**Affiliations:** 1 Internal Medicine, Jawaharlal Nehru Medical College, Datta Meghe Institute of Higher Education and Research, Wardha, IND; 2 Obstetrics and Gynecology, Jawaharlal Nehru Medical College, Datta Meghe Institute of Higher Education and Research, Wardha, IND

**Keywords:** atypical hemolytic uremic syndrome (ahus), hip arthroplasty, myositis ossificans, plasmapheresis, renal dysfunction, thrombocytopenia

## Abstract

Atypical hemolytic uremic syndrome (aHUS) is a rare and complex disease characterized by microangiopathic hemolytic anemia, thrombocytopenia, and acute renal failure. This case report details the clinical presentation, diagnosis, and management of a 49-year-old female who developed aHUS following elective hip arthroplasty. The patient, with a history of cardiovascular events and no prior renal disease, presented with elevated LDH levels, thrombocytopenia, and acute renal failure on the first postoperative day. A diagnostic workup confirmed aHUS, and the patient was successfully treated with therapeutic plasma exchange (TPE) and hemodialysis. The case underscores the importance of early recognition and aggressive management of aHUS, especially in the perioperative setting, and highlights the need for a multidisciplinary approach to optimize patient outcomes. Through this case, we aim to raise awareness about the potential for surgical stress to trigger aHUS and emphasize the critical role of TPE and supportive care in the treatment of this rare condition.

## Introduction

Atypical hemolytic uremic syndrome (aHUS) is a rare, life-threatening condition characterized by microangiopathic hemolytic anemia, thrombocytopenia, and renal failure. Unlike typical HUS, which is often associated with Shiga toxin-producing *Escherichia coli* (STEC), aHUS is frequently linked to dysregulation of the complement system [1]. Genetic mutations in complement regulatory proteins such as complement factor H (CFH), membrane cofactor protein (MCP or CD46), and complement factor I (CFI) are commonly implicated in the pathogenesis of aHUS [2]. The incidence of aHUS is approximately two per million per year, with a predilection for females and a peak incidence in childhood and middle age [3]. Various factors, including infections, pregnancy, malignancies, and certain medications can trigger the condition. In the context of surgical interventions, such as hip arthroplasty, the stress of surgery and perioperative management can precipitate an aHUS episode [4].

The management of aHUS primarily involves using eculizumab, a monoclonal antibody that inhibits the terminal complement pathway, and supportive therapies, including plasmapheresis (therapeutic plasma exchange (TPE)) and dialysis [5]. Early diagnosis and prompt initiation of therapy are crucial to improving outcomes and preventing irreversible organ damage [6]. This case report describes a 49-year-old female who developed aHUS following elective hip arthroplasty. The patient's medical history, clinical presentation, diagnostic workup, and therapeutic management are detailed, highlighting the complexities of diagnosing and treating aHUS in the perioperative setting.

## Case presentation

A 49-year-old female was admitted to our hospital with complaints of left hip joint pain. A computed tomography (CT) scan of the pelvis revealed a large, ill-defined, irregular exophytic bony outgrowth (osteoma) with heterogeneous mineralization. This outgrowth originated from the anterior aspect of the left proximal femoral diaphysis and extended superiorly to the iliac bone. A similar bony outgrowth (osteoma) was noted arising from the inner aspect of the iliac blade on the left side, extending antero-inferiorly to form pseudoarthrosis with the osteoma of the left femur.

**Figure 1:**
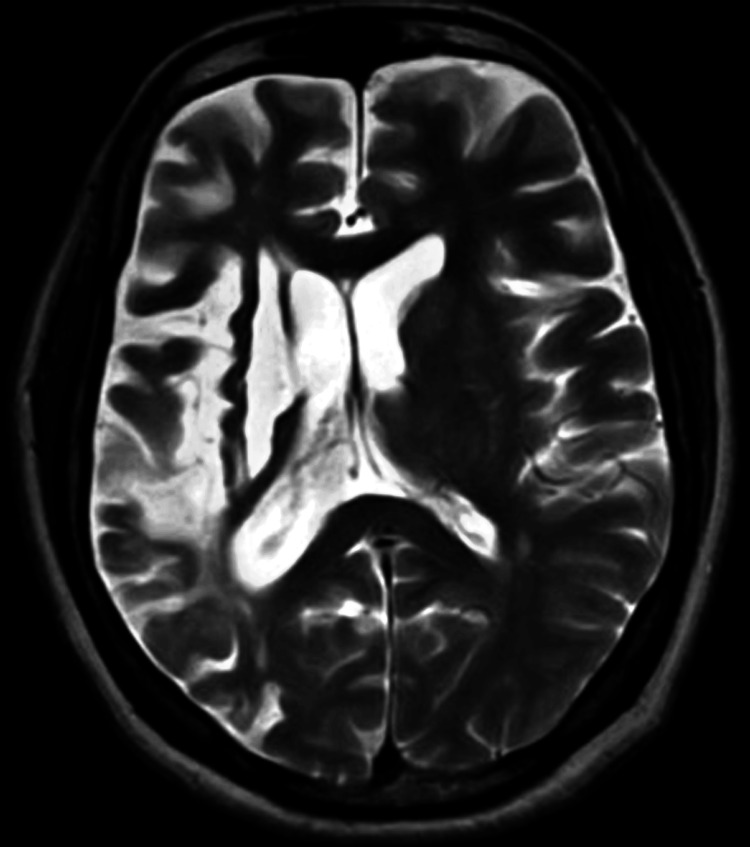
Magnetic resonance imaging (MRI) of the brain indicated an acute infarct in the right fronto-parietal-temporal lobe and gangliocapsular region (right middle cerebral artery (MCA) territory)

The patient had a history of a cardiovascular event (CVE) three years ago. At that time, magnetic resonance imaging (MRI) of the brain indicated an acute infarct in the right fronto-parietal-temporal lobe and gangliocapsular region (right middle cerebral artery (MCA) territory), with non-visualization of the M2, M3, and M4 segments of the right MCA, possibly due to thrombosis (Figure 1). She was managed conservatively and advised to undergo physiotherapy. She reported a fall while performing physiotherapy two months after its initiation and was managed conservatively, continuing with physiotherapy after that. She had no known history of autoimmune disorders or renal diseases. The patient underwent surgery for the bony outgrowths, and the excised tissue was sent for histopathological examination, which suggested myositis ossificans. Before this intervention, her laboratory test results were unremarkable, with normal creatinine, platelet count, and hemoglobin values.

During the procedure performed under spinal and epidural anesthesia, minimal blood loss occurred. She was prescribed aspirin twice a day to prevent venous thromboembolism. Her vital signs were stable postoperatively. A complete blood count follow-up was conducted on the first and second postoperative days. Day 1 results revealed increased LDH levels (714), decreased platelet count to 114,000, increased creatinine to 7.4, and hemoglobin at 9.7 g/dL (mild anemia). These findings led to a diagnosis of aHUS. A peripheral smear confirmed moderate thrombocytopenia and fragmented red blood cells. Preoperative and postoperative investigations are detailed in Table 1, with other investigations in Table 2.

**Table 1:** Preoperative and postoperative investigations

Investigations	Normal range	Preoperative values	Postoperative values (day 1)	Postoperative values (day 7)	Postoperative values (day 14)	Values on discharge
Hemoglobin (gm%)	12-16	10.9	9.7	8.6	9.5	9.7
White blood cells (/mm^3^)	4,500 to 11,000	7400	11500	10800	11000	8800
Platelets (L/cumm)	1.5 to 4	2.96	1.14	0.41	0.97	1.03
Urea (mg/dL)	Male: 9 to 20; female: 7 to 17	15	112	184	117	43
Creatinine (mg/dL)	Male: 0.6 to 1.25; female: 0.52 to 1.04	1.1	7.4	11.7	6.6	1.7
Lactate dehydrogenase (LDH) (IU/L)	120 to 246		714	486	286	267

**Table 2:** Other investigations performed

Investigations	Normal range	Values
C-reactive protein (mg/dL)	<1.0	30.313
Complement component 3 (C3) (mg/dL)	80 to 165	40
Complement component 4 (C4) (mg/dL)	14 to 44	42
Anti-nuclear antibodies (ANA)	Less than 0.9: negative; 0.9 to 1.1: borderline positive; more than 1.1: positive	1.11

**Figure 2:**
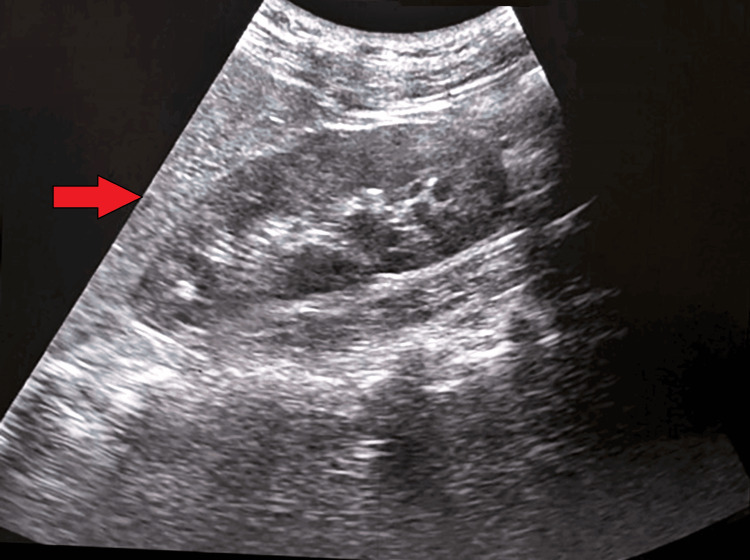
Ultrasonography of the kidneys, ureters, and bladder (USG-KUB) suggested grade 1 renal parenchymal disease (arrow)

Ultrasonography of the kidneys, ureters, and bladder (USG-KUB) suggested grade 1 renal parenchymal disease (Figure 2). A nephrology consultation was obtained for further management, and the patient was advised to undergo alternate plasmapheresis, also known as TPE and dialysis. Following blood grouping and cross-matching, she underwent six cycles of plasmapheresis and three cycles of hemodialysis. She received 82 fresh frozen plasma (FFP) units and two packed red cells (PRC) transfusions.

Due to sepsis, blood and urine cultures were performed, and antibiotics were administered accordingly. The patient was advised to maintain adequate hydration, monitor daily fluid intake and output, and regularly check blood pressure, oxygen saturation (SpO_2_), and random blood sugar (RBS). Symptomatic treatment was provided as needed. Throughout her stay, the patient remained vitally stable. ADAMST13 activity was found to be above 50%, which is normal. The Shiga toxin fecal polymerase chain reaction test was negative, confirming the diagnosis of atypical HUS.

**Figure 3:**
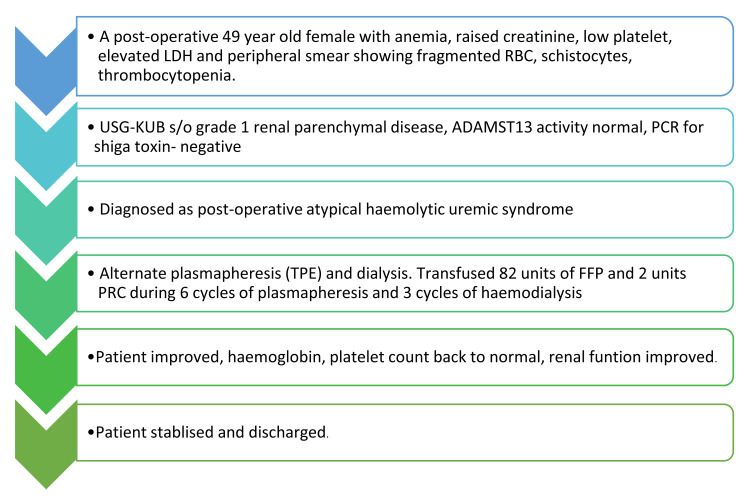
Clinical course of the patient in the hospital LDH: lactate dehydrogenase; USG-KUB: ultrasonography of the kidneys, ureters, and bladder; PCR: polymerase chain reaction; FFP: fresh frozen plasma;

The patient's clinical condition gradually improved after the TPE and supportive therapy. Her hemoglobin and platelet count normalized, and renal function showed signs of improvement. Repeat urine analysis indicated a resolution of proteinuria and hematuria. The patient was discharged from the hospital after close follow-up with the nephrology team for ongoing management and renal function monitoring. The clinical course is depicted in Figure 3.

## Discussion

aHUS is a rare, life-threatening disease characterized by microangiopathic hemolytic anemia, thrombocytopenia, and acute renal failure. The pathophysiology of aHUS involves dysregulation of the complement system, leading to uncontrolled complement activation and subsequent endothelial damage, platelet activation, and thrombus formation. This case illustrates the diagnostic and therapeutic challenges of aHUS in a patient undergoing elective hip arthroplasty. The patient's history of CVEs, including an acute infarct in the right MCA territory, underscores the complexity of her medical background. The presentation of aHUS in the postoperative period is consistent with previous reports that suggest surgical stress, infections, and other systemic insults can trigger aHUS episodes [1,7]. In this case, the patient developed aHUS symptoms, including elevated LDH levels, thrombocytopenia, and acute renal failure, on the first postoperative day. These findings align with the typical clinical presentation of aHUS, where rapid deterioration of renal function and hematological abnormalities are observed [8]. Early recognition and diagnosis were crucial in managing this condition, as delays can lead to irreversible renal damage and other severe complications [5]. This patient's use of TPE and hemodialysis was pivotal in her management. TPE effectively removes circulating autoantibodies, mutant complement factors, and other pathogenic substances, thus reducing the severity of the disease [9]. Additionally, administering FFP during TPE sessions provided essential complement regulatory proteins, further aiding disease control [10].

The patient's response to TPE and supportive therapy, evidenced by the normalization of hemoglobin and platelet counts and improvement in renal function, underscores the importance of early and aggressive treatment. The use of multiple cycles of TPE and hemodialysis is consistent with established treatment protocols for aHUS [11,12]. Moreover, the negative results for Shiga toxin and the normal ADAMTS13 activity excluded other potential causes of hemolytic uremic syndrome (HUS), such as STEC infection and thrombotic thrombocytopenic purpura (TTP), respectively [13]. These findings further supported the diagnosis of aHUS. Sepsis management was another critical aspect of this patient's care. Blood and urine cultures and appropriate antibiotic therapy were necessary to address any potential infections that could exacerbate the patient's condition. Ensuring adequate hydration, monitoring fluid intake and output, and maintaining stable vital signs were essential components of the supportive care provided [14]. This case highlights the need for heightened awareness and prompt intervention in patients at risk for aHUS, particularly in the perioperative setting. Multidisciplinary collaboration involving nephrology, hematology, and critical care teams is essential for optimizing patient outcomes. Future research should focus on identifying predisposing factors and developing targeted therapies to prevent and treat aHUS more effectively.

## Conclusions

This case report underscores the complexity and severity of aHUS, especially in the context of postoperative complications following elective hip arthroplasty. The rapid onset of aHUS symptoms postsurgery highlights the importance of vigilant monitoring and early recognition of this rare condition. The effective use of TPE and hemodialysis was crucial in managing the patient's condition, demonstrating the need for prompt, aggressive intervention to prevent irreversible damage. Multidisciplinary collaboration and thorough diagnostic workup were key in ruling out other potential causes and ensuring appropriate treatment. This case emphasizes the importance of considering aHUS in patients presenting with hemolytic anemia, thrombocytopenia, and renal dysfunction, particularly in those with a history of CVEs or surgical interventions. Future advancements in understanding the pathophysiology of aHUS and the development of targeted therapies will be vital in improving patient outcomes.
